# Boron Neutron Capture Therapy: Current Status and Challenges

**DOI:** 10.3389/fonc.2022.788770

**Published:** 2022-03-31

**Authors:** Song Wang, Zhengchao Zhang, Lele Miao, Yumin Li

**Affiliations:** ^1^Department of General Surgery, Second Hospital of Lanzhou University, Lanzhou, China; ^2^Key Laboratory of the Digestive System Tumors of Gansu Province, Second Hospital of Lanzhou University, Lanzhou, China

**Keywords:** boron neutron capture therapy (BNCT), thermal neutron, boron delivery agent, radiation, tumor

## Abstract

Boron neutron capture therapy (BNCT) is a re-emerging therapy with the ability to selectively kill tumor cells. After the boron delivery agents enter the tumor tissue and enrich the tumor cells, the thermal neutrons trigger the fission of the boron atoms, leading to the release of boron atoms and then leading to the release of the α particles (^4^He) and recoil lithium particles (^7^Li), along with the production of large amounts of energy in the narrow region. With the advantages of targeted therapy and low toxicity, BNCT has become a unique method in the field of radiotherapy. Since the beginning of the last century, BNCT has been emerging worldwide and gradually developed into a technology for the treatment of glioblastoma multiforme, head and neck cancer, malignant melanoma, and other cancers. At present, how to develop and innovate more efficient boron delivery agents and establish a more accurate boron-dose measurement system have become the problem faced by the development of BNCT. We discuss the use of boron delivery agents over the past several decades and the corresponding clinical trials and preclinical outcomes. Furthermore, the discussion brings recommendations on the future of boron delivery agents and this therapy.

## Introduction

**Figure 1:**
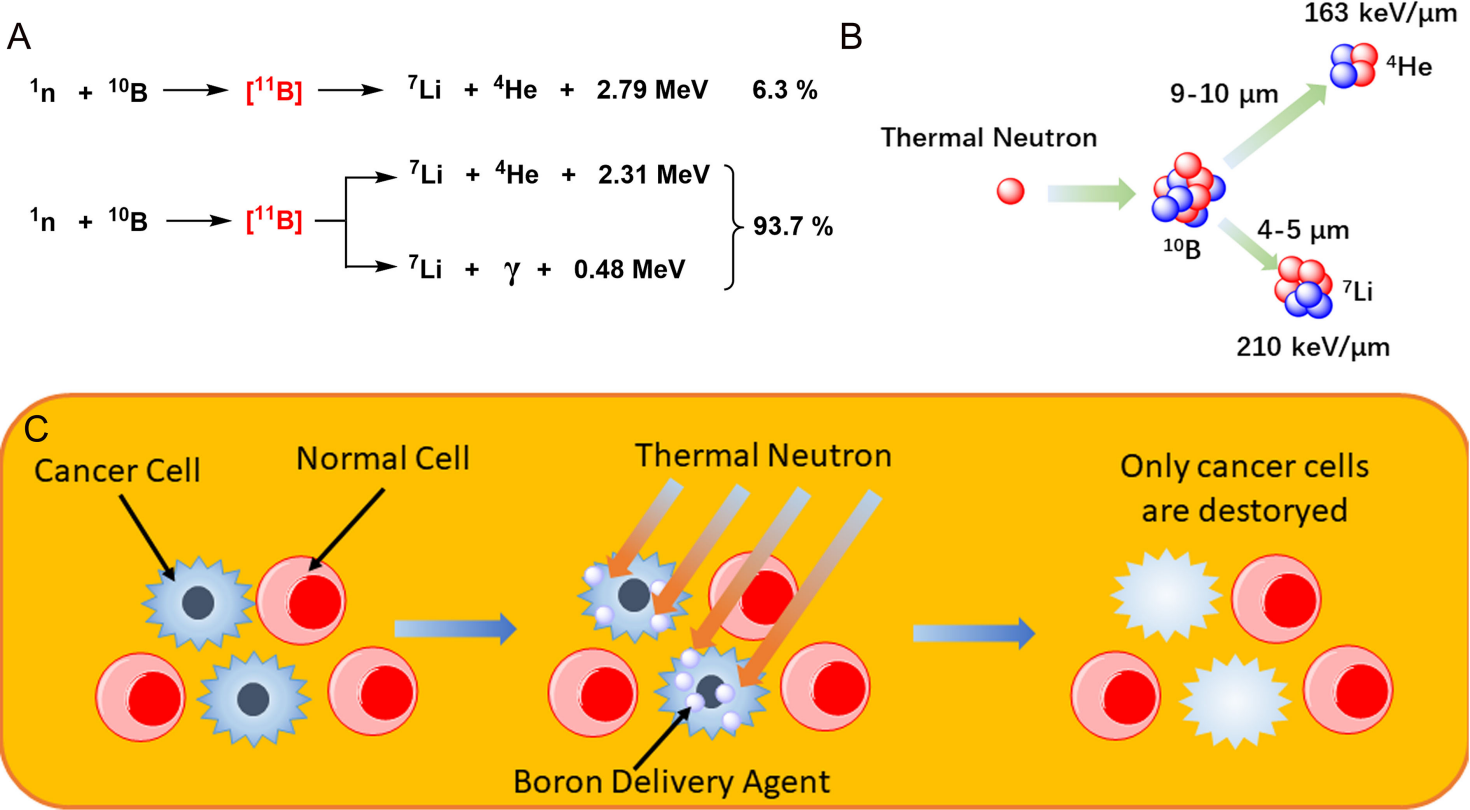
**(A)** The nuclear reaction of boron atom, the dominant of which is accompanied by the production of high-energy rays. **(B)** Mechanism of boron neutron capture therapy. **(C)** Schematic diagram of boron neutron capture therapy (BNCT) selective killer tumor cells.

The role of radiation therapy in the treatment of cancer is widely recognized, and radiation therapy is required in at least 50% of cancer patients (1). Although radiotherapy has long been considered a local treatment, it is likely to lead to serious complications, especially in patients with recurrent cancer, which will make the symptoms more complex and difficult to cure. With the development of biological and physical technologies, researchers have developed a number of new approaches that may be used to solve these problems. One of them is boron neutron capture therapy (BNCT), a technology based on boron nuclear reactions (**Figure 1A**). After being irradiated by neutrons, ^10^B becomes unstable ^11^B, which continues to decay its energy and radiates α particles (^4^He) and ^7^Li recoil particles, releasing a large amount of energy and accompanied by a small amount of gamma rays during the reaction. The BNCT treatment process requires selectively boron accumulation of non-radioactive drugs in tumor cells, followed by the irradiation of local tissue with neutron beams, causing the release of large quantities of high-energy particles with nuclear fission to selectively kill the tumor cells in a narrow range (**Figure 1B**). The radiation radius of the reaction limits its scope of action, and tumor cells can be rapidly and selectively killed once boron is ingested without damaging the surrounding normal tissue (2, 3) (**Figure 1C**). BNCT has been studied in a variety of diseases, including glioblastoma multiforme (GBM), primary and recurrent head and neck cancer, lung cancer, liver cancer, and extramammary Paget’s disease (4, 5). Therefore, BNCT has very good development value and application prospects (**Table 1** summarizes the clinical trials of BNCT in recent years). In fact, BNCT has been evaluated as an alternative treatment for a variety of cancers, including GBM, melanoma, and head and neck cancer in multiple phases I and II clinical trials. However, there are many factors against BNCT, such as cell selectivity of boron delivery agents, delivery mode, and irradiation depth of neutron beam and reactor, all of which involve the intersection of multiple technologies.

**Table 1:** Clinical trials of different boron delivery agents.

Tumor type	Number of patients	Boron delivery agent
Glioblastoma	269	BPA, BPA-F, BSH, BPA/BSH with ERBT
Head and neck	30	BPA-F
Recurrent head and neck	215	BPA-F, BSH, BPA/BSH
Melanoma	67	BPA-F
Lung cancer	1	BPA-F
Liver metastases of colorectal adenocarcinoma	2	BPA, BSH
Extramammary Paget’s disease	2	BPA-F

The data were collected from Helsinki University Central Hospital, the Third Xiangya Hospital of Central South University, Taipei Veterans General Hospital, Taiwan, European Organisation for Research and Treatment of Cancer, Cancer Intelligence Care Systems, Inc., Translational Research Center for Medical Innovation, the U.S. National Library of Medicine, the U.S. National Institutes of Health, and the U.S. Department of Health and Human Services.

BPA, para-boron-phenylalanine; BPA-F, para-boron-phenylalanine-fructose; BSH, borocaptate sodium; ERBT, external beam radiation therapy.

## Clinical Investigations

Considering the factors affecting the application of BNCT, it is necessary to pay attention to the key issues at each stage of the design, such as the location and depth of the lesion, the absorption of the ^10^B delivery agent, the cycle and intensity of neutron beam irradiation, and the pharmacokinetics. Previously, Joensuu and Hideghéty et al. from the University of Helsinki have done detailed studies on the metabolism and toxicity of several boron delivery agents (6–8). The concentration and injection rate of *para*-boron-phenylalanine (BPA)-fructose (BPA-F) were controlled below 500 mg/kg/h, and a large number of relevant clinical data were obtained. These results suggest the feasibility of this therapy and provide valuable experience for further exploration. As we can see from **Table 1**, BNCT is still not widely used, restricted by insufficient experience in drug usage, difficulties in patient recruitment, lack of medical neutron sources, and other factors; this has resulted in relatively slow development and clinical implementation of BNCT. We will discuss these issues in detail in this review.

Compared with the traditional therapy, BNCT has the following advantages (9): a) the level of boron-containing drugs in tumor cells differs greatly from that in normal cells, leading to the content of ^10^B in tumor cells being higher than that in normal cells, so the damage to normal cells is less; b) radiotherapy requires oxygen to enhance the effect of biological radiation, but the aggressive proliferation of malignant tumor cells is very fast, which causes local hypoxia and reduces the therapeutic effect. Moreover, α particles and ^7^Li are not dependent on oxygen, so they have the same ability against tumor cells in oxygen-rich and hypoxic environments. c) Chemotherapy, γ knife, and radiotherapy have effects on tumor cells in the proliferative cycle (G1, S, G2, and M stages) but are not sensitive to G0 phase tumor cells. However, α particles and ^7^Li killer cells are independent of the cell cycle and can kill tumor cells at all phases. This review will introduce the research status of BNCT, the design and study of boron drugs, the results of clinical trials, and the therapeutic characteristics.

Based on previously published results (10–12), we focus on clinical trials over the past 20 years. Locher first elaborated the idea and principle of neutron capture therapy (NCT) in 1936 (13). The first clinical trial of BNCT was initiated by Farr and Sweet et al. in 1951. They used Brookhaven Graphite Research Reactor to treat GBM (14). In 1963, Sweet et al. used *para*-carboxylic phenylboric acid and Na_2_B_10_H_10_ as boron delivery agents for BNCT treatment in glioma patients. Na_2_B_10_H_10_ demonstrated better efficacy in the experiment, including lower toxicity and a higher ^10^B distribution ratio (tumor/blood) (15). By 1968, Hatanaka et al. showed a 5-year survival rate of 58% when they pretreated the patient’s tumor tissue with borocaptate sodium (BSH) (Na_2_B_12_H_11_SH) and directly irradiated the surgically exposed intracranial tumor bed with a neutron beam, a very good result for that day (15–17). In 1987, Mishima and his colleagues first used BNCT in the treatment of malignant melanoma *via* BPA as a boron delivery agent. It was also the first attempt to use BNCT in the treatment of peripheral tumors outside the central nervous system.

Until the 1990s, BNCT research was intensive. Since then, several countries have suspended BNCT research, which has been linked to incidents in some nuclear reactions. After that, the work related to BNCT has focused on the pharmaceutical properties of boron delivery agents and animal experiments. However, in Taiwan Veterans General Hospital, clinical research related to BNCT on head and neck cancer was carried out in 2010 and 2013. They increased the dose to 500 mg/kg and supplemented it with superficial irradiation, which also achieved ideal effects. In recent years, The Third Xiangya Hospital of Central South University in China conducted a BNCT study involving 22 melanoma patients from 2013 to 2016. They used a specialized in-hospital neutron irradiator (IHNI) and continued to deliver BPA-F at a speed of 180 mg/kg/h. The results showed that within 24 months after the treatment with this therapy, the pigment plaque produced by the lesions in the affected area was significantly less. A small amount of residual can be identified as small pigmented plaque formed by the deposition of dead cells. The results of case analysis conducted about 9 months after treatment also indicated that these small pigmented plaques did not spread and did not invade deep cortical layers, causing metastasis and recurrence, which is a very exciting result (18). Here we can see that there is still a vast space for the development and clinical application of boron delivery agents.

Boron-containing compounds have been extensively studied in many areas including pharmaceutical chemistry and medicine. While the value of boron-containing compounds has long been recognized, there are still only a few boronated drugs to choose from. Carboranes are a class of organic compounds containing carbon, boron, and hydrogen. They are the most widely studied boron compounds in pharmaceutical chemistry. In addition, other boron-containing compounds are also of great concern, such as the perhydrodecaborate anion. BNCT has been used for cancer research for decades; before the full clinical potential of BNCT can be realized, there is a need to improve existing topical delivery agents (BPA/BSH) or develop some novel tumor-targeting boron delivery agents. Here we present the current clinical trials of boronated drugs (**Table 2**) and focus on the improvement and clinical significance of developed and improved boron-based bioactive compounds in the treatment of BNCT.

**Table 2:** Clinical trials of different boron compounds.

Tumor type	Boron delivery agent	Country or institution	Date	Reference
**Glioblastoma**	^10^B	USA	1951–1953	(14)
**Glioblastoma**	*p*-Carboxy derivative of phenylboronic acid	USA	1959–1961	(19)
**Glioblastoma**	BPA-F	Finland	1999–2012	(8)
**Glioblastoma multiforme**	BSH	Czech Republic	2000–2002	(20, 21)
**Glioblastoma multiforme**	BPA	Sweden	2001–2003	(22)
**Glioblastoma multiforme**	BPA-F	Sweden	2000–2003	(23)
**Malignant gliomas**	BPA/BSH	Japan	2002–2003	(24, 25)
**Malignant gliomas**	BSH	Japan	2004–	(26)
**Malignant gliomas**	BPA-F	Finland	2001–2008	(27)
**Recurrent malignant glioma**	BPA	Japan	2002–2007	(28, 29)
**Meningiomas**	BPA/BSH	Japan	2004–2006	(30, 31)
**Head and neck**	BPA/BSH	EORTC	1996–2007	(32, 33)
**Head and neck**	BPA-F	Finland	2001–2009	(34)
**Head and neck**	BPA	Taiwan	2010–	(35, 36)
**Head and neck**	C-BENS with borofalan (^10^B)	Japan	2021	(37)
**Head and neck**	BPA	Taiwan	2021	(38)
**Melanoma**	BPA	Japan	1987–	(39)
**Melanoma**	BPA	Argentina	2003–2007	(40)
**Melanoma**	BPA-F	China	2013–	(18)
**Liver metastases of colorectal adenocarcinoma**	BPA/BSH	Germany	2004–	(41)
**Hepatocellular carcinoma**	BSH	Japan	2011–	(42)
**Extramammary Paget’s disease**	BPA-F	Japan	2003–2014	(43)

BPA, para-boron-phenylalanine; BPA-F, para-boron-phenylalanine-fructose; BSH, borocaptate sodium; C-BENS, cyclotron-based epithermal neutron source; EORTC, European Organisation for Research and Treatment of Cancer.

One advantage of BNCT over conventional radiotherapy is that its clinical efficacy depends largely on the high accumulation of ^10^B in tumor cells with minimal uptake of normal cells. This would allow the BNCT to target cancer cells for damage while protecting the surrounding normal tissue. Therefore, the development of boron delivery agents with high selectivity is one of the main directions in the development of BNCT. Based on clinical experience during the development of the BNCT, the researchers summarized a number of principles for evaluating the efficacy of the delivery agent. The five most important factors are as follows: each tumor cell contains more than 10^9^ (1.66 × 10^−3^ pmol) ^10^B atoms; the T/N (tumor/normal) and T/B (tumor/blood) concentration ratios of boron atom are greater than or equal to 3; the drug itself has low cytotoxicity; it can be quickly removed from normal tissues and blood but has a long time of stable retention in tumor cells; it is distributed in lipophilic and hydrophilic phases (amphiphilicity of delivery agents) in tissue, especially in brain tumors (2, 10, 44, 45). These principles guide the design, selection, and development of reagents for use in BNCT for subsequent *in vitro* and *in vivo* evaluations. According to the types of carriers, boron delivery agents could be divided into three types: boron-containing small molecules, boron-containing complexes, and boron-containing nanoparticles (2, 44, 46–49). Among these vectors, the most interesting development direction is targeted boron delivery agents, which usually bind boron-containing agents to various targeted molecules (such as nucleosides, peptides, proteins, porphyrins, or antibodies) of tumor cells. Another targeted boron delivery agent is the combination of boron-containing molecules and nanomaterials, which can take advantage of the enhanced permeability and retention (EPR) effects of nanomaterials as well as the active targeting effect mediated by grafted tumor-targeting ligands to transport boron-containing compounds into tumor cells (49).

## Clinical Trials With Boron Delivery Agents

### Boron Delivery Agents

BNCT is a novel method, which is still being tested, and its potential is clear, though it will have little impact on the mainstream of medicine. Chadwick discovered the neutron in 1932, and 4 years later, Locher proposed using the neutron capture reaction in cancer treatment (13). The first clinical trial of BNCT was not implemented until 1951 (14), and in 1961, about 40 patients were treated with BNCT in 10 years with varying degrees of results, but often with varying degrees of side effects (50), such as necrosis (51), edema, and refractory shock (52). These disappointing results led the BNCT study to stop in the United States. In 1968, Hatanaka conducted the BNCT trial in Japan, using the synthetic compound BSH as a boron delivery agent for treatment as intraoperative radiotherapy after tumor resection (53), where direct exposure of the tumor bed to a neutron beam reported encouraging results, with a 5-year overall survival (OS) rate of 58% in patients with grade 3 and 4 GBM (16, 17, 54). By 1987, Mishima et al. began treating superficial malignant melanoma with BPA. This section focuses on the relatively complete clinical trials of BNCT and related boron delivery agents, with details on GBM, head and neck cancer, and melanoma (39, 55).

**Figure 2:**
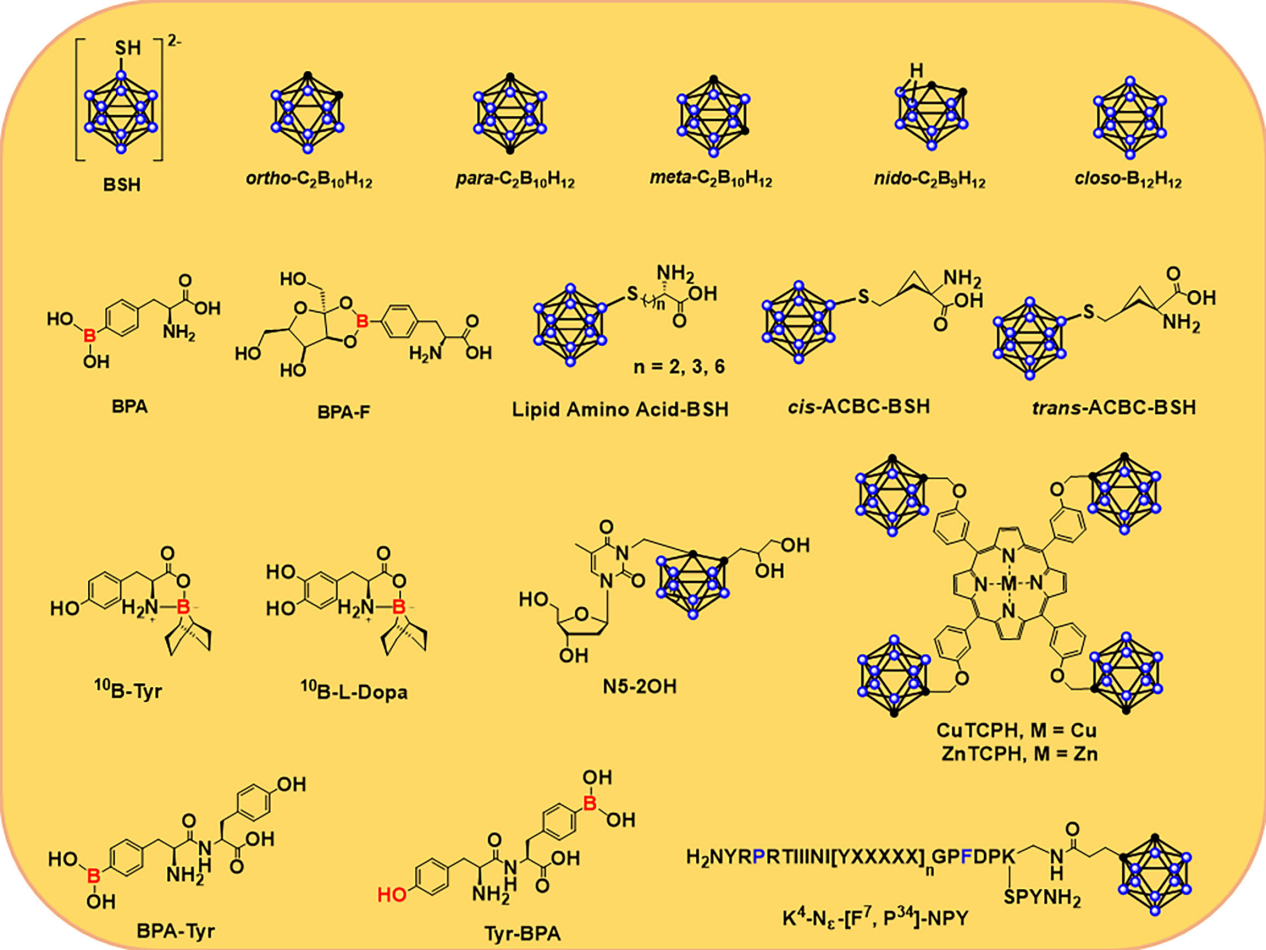
Structures of several ^10^B delivery agents, including carboranes and carboranes (39, 55, 56) that are conjugated to nucleosides (57–59), porphyrins (60), amino acids (11, 61, 62), and peptides (63).

**Figure 2** shows a variety of modified small molecule boron agents, some of which have been in clinical trials for many years, while others are still under study. At present, only BPA (64) and BSH (53) have been applied in clinical trials, among which BSH is the first boron-containing compound in clinical application. Its advantage lies in its extremely high boron content; that is, each BSH molecule carries 12 ^10^B atoms, which is very helpful for the accumulation of ^10^B in tumor cells. This molecule was synthesized by Hatanaka et al. It was used to treat glioma in 1968 (39). In 1987, Mishima et al. first used BPA to treat malignant melanoma and found that it can selectively accumulate in tumor cells (55, 56). BPA is a phenylalanine analog, which is mainly transported to tumor cells through L-type amino acid transporter 1 (LAT-1) on the surface of the cell membrane, and this protein is overexpressed on the surface of malignant tumor cells. Hence BPA is also easier to accumulate in tumor cells. In clinical trials, BPA has a better affinity for tumor cells than BSH, and the emergence of BPA has also significantly accelerated the progress of research on targeted boron delivery agents.

BPA-F has better solubility than BPA; fructose is linked to BPA in the form of borate ester, resulting in improved solubility and biocompatibility of BPA. In addition, this combination promoted the more efficient distribution of BPA in the body and increased uptake by tumor cells. In 1994, BNCT was used for malignant intracranial glioma *via* BPA-F (65). Since then, BPA-F has been widely used in clinical trials in Japan, Sweden, Finland, China, and Taiwan to treat a variety of malignancies. Hattori et al. synthesized a series of derivatives of BSH (**Figure 2**) with low cytotoxicity and high lipophilicity and thus could be used as novel boron delivery agents in BNCT (61, 66). Compared to BPA alone, these delivery agents showed higher uptake and kill rates on cells (66). They found that the α,α-cycloalkyl amino acids, known as cis- or trans-ACBC, caused tumor cells to accumulate more ^10^B atoms than L-BPA and its short-chain lipid amino acid derivatives in several cell lines (C6, U81, and A172) expressing the L-type amino acid transporter (LAT) (61, 62). Al-Madhoun et al. analyzed the properties of 3-carboranyl thymidine analogs (3-CTA), including catalytic kinetic parameters, stability against other enzymes, *n*-octanol/water distribution coefficient, cytotoxic activity, intake of compounds, and retention time in various cell lines (57, 67). At last, they study the distribution of ^10^B atoms in rats with F98-glioma *via* N5-2OH (57). The results indicated that the level of ^10^B in glioma and normal brain tissue was 16.2 ± 2.3 and 2.2 μg/g, respectively. The concentration ratio of ^10^B in glioma and normal brain tissue was 8.5 (57, 68).

Miura et al. synthesized a series of complexed metallic porphyrins (60, 69–74). Cu(II) and Zn(II) porphyrins complex, also known as CuTCPH and ZnTCPH in **Figure 2**, are currently the most deeply studied porphyrin derivatives *in vitro* and *in vivo* (60). After injection of ZnTCPH or CuTCPH into mice, the distributions of ^10^B in different tissues were similar in mice carrying EMT-6, and the T/B and T/N ratios of ^10^B were larger (about 60:1 and 8:1, respectively). The tumor-bearing mice were injected with ZnTCPH fluorescence in the spleen, liver, and tumor. CuTCPH or ZnTCPB can also be used as a probe for tumor imaging *via* ^67^Cu-based single photon-emission CT (SPECT) or ^64^Cu-based positron emission tomography (PET) (60).

Peptide transporter 1 (PepT1) is an oligopeptide transporter whose expression is increased in tumor cells. As a potential biomarker and tumor therapeutic target, PepT1 has attracted much attention (75, 76). Miyabe et al. proposed the selective delivery of boron to tumor cells *via* PepT1. They found that the dipeptide (BPA-Tyr and Tyr-BPA, **Figure 2**) formed by condensation of BPA and tyrosine could be ingested by ASPC-1 cells *via* the PepT1-mediated oligopeptide transport pathway; their T/B ratios of ^10^B were 2.00 ± 0.09 and 1.98 ± 0.05, respectively, 3 h after the drug was injected into AsPC-1-bearing mice. At 3 and 5 h after injection, the concentrations of ^10^B were 7.29 ± 0.31 and 6.48 ± 0.30 ppm, respectively (77).

Due to the ability of neuropeptide Y (NPY) to bind to the Y receptor with high affinity, the researchers developed a number of boron-containing NPY complexes as boron delivery agents based on this. [F^7^, P^34^]-NPY showed a high affinity for the hY1 receptor (78), which led Ahrens et al. to develop *ortho*-carborane-containing NPY and a series of derivatives. The L-Lys modified by *ortho*-carboane was conjugated with [F^7^, P^34^]-NPY to obtain [K^4^-Nε(Cpa)]-[F^7^, P^34^]-NPY (**Figure 2**), which makes this molecule have extremely high affinity even at the nanomolar level and easy to be selectively ingested by tumor cells of the corresponding subtype (63).

In 2005, Wong et al. used cetuximab (IMC-C225, Erbitux) as an antibody against EGFR-mAbs for EGFR-positive recurrent colon cancer (79). Then, Wu et al. condensed cetuximab with the amino group of boronated polyamide dendrimer to obtain the bioconjugated BD-C225, which was absorbed by F98EGFR glioma and remained in the tumor cells for a long time, with undetectable ^10^B concentrations in blood and normal tissues (80, 81). They bonded cetuximab to Mal-PEG-Chol (maleimide-PEG-cholesterol liposomes) assembled with closo-carborane to enhance the drug uptake in F98EGFR gliomas by using the affinity of liposome to cell membrane structures (82). Yang et al. bonded modified cage boranes with polyamide amine dendrimer to form boronated dendrimer (BD). In addition, L8A4BD was re-bonded by L8A4 as a specific monoclonal antibody of EGFRVIII. The obtained molecule not only has the EGFRVIII targeting ability but also possesses the ^10^B to tumor cells and can also be used as an antibody–drug conjugate (83).

For boron delivery agents, targeting ability is the most critical factor. In addition to the targeting ability of the molecule to the tumor, the groups connected can improve the targeting ability of delivery agents to different cells. The most important criterion is the distribution of boron in the tumor and normal tissues. Besides, even in the case of more novel structural designs, the need to find molecules with potential effects *via* high-throughput screening ultimately requires strong coordination of synthetic chemistry. From the current development of boron drugs, researchers have found some boron-containing molecules as delivery agents, and there are fewer complications caused by the toxicity of the boron delivery agents in clinical trials. However, the targeting of molecules is not satisfactory at present, and there are still cases where the difference of boron concentration in tumor tissue is close to that in blood, skin, and mucosal tissue. Therefore, a future focus should be on chemical synthesis to improve target uptake of the molecules.

### Glioblastoma

GBM is a common and aggressive malignant brain tumor. Currently, treatment for the disease is typically a multimodal combination of surgical resection, radiation, and chemotherapy. Conventional methods have a poor prognosis for high-grade gliomas, although radiation therapy is used to minimize tumor regression. Therefore, the inclusion of BNCT in combination therapy may be helpful for the cure of GBM. **Table 3** summarizes relatively complete clinical studies of BNCT in GBM from 1999 to date.

**Table 3:** Clinical studies of BNCT application in glioblastoma.

Institution	Treatment dates	Number of patients	Boron compound and treatment	Clinical outcome	Phase
Department of Oncology, University of Helsinki (8, 84)	1999	72	BPA-F 290~500 mg/kg; 1.8~2.0 Gy/day	1-year OS: 61%	I/II
European Organisation for Research and Treatment of Cancer (6, 7, 52, 85, 86)	2002	36	BSH 100 mg/kg;	1-year OS: 50%	I
Translational Research Center for Medical Innovation (87)	2009	32	BSH 100 mg/kg; BPA 200 mg/kg; 2.0 Gy/day	MST: 15.6 months	I/II
Kagawa National Children’s Hospital, Japan (88)	2011	23	BSH 100 mg/kg; BPA 250 mg/kg; 18.375.3 Gy	MST: 26.2 (NO-BNCT) months	I/II

BPA-F, para-boron-phenylalanine-fructose; BSH, borocaptate sodium; OS, overall survival; MST, median survival time. NO-BNCT, non-operative BNCT.

Since 1999, Joensuu et al. have carried out clinical studies of BNCT for malignant glioma. In the first clinical trial, a total of 18 patients with GBM received BNCT based on a derivative of BPA. All patients had undergone surgery before BNCT, but none had undergone radiotherapy. The results showed that the treatment was generally well tolerated, with no patients dying in the months following and an overall 1-year survival rate of 61%. In another trial, none of the three patients with recurrent GBM died in the 3 months following BNCT (8). At the same time, Hideghéty et al. conducted clinical trials to study the uptake of the boron compound BSH in tumor and normal tissue. They measured levels of ^10^B in the blood, tumors, brain tissue, dura, muscle, skin, and bone of 13 patients who received the treatment. In the first group (10 patients), BSH was administered at 100 mg/kg; three patients in the second group received BSH at a concentration of 22.9 mg/kg. The results suggested that the mean boron concentration in tumor cells was 19.9 ± 9.1 ppm in the first group and 9.8 ± 3.3 ppm in the second group, and their T/B distribution ratios of ^10^B were 0.6 ± 0.2 and 0.9 ± 0.2, respectively. Among other tissues, dura absorption was the highest, and boron absorption was very low in the bone, cerebrospinal fluid, and especially the brain (brain/blood distribution ratios of ^10^B in both groups were 0.2 ± 0.02 and 0.4 ± 0.2). Therefore, BSH can be safely administered in the clinic at rates of 100 mg/kg/min. The study also speculated that boron concentration in the blood appeared to be a very reliable parameter for predicting boron concentration in other tissues.

Verbakel et al. conducted a dose-escalation research *via* BSH. The results indicated that the ratio of boron in blood to normal brain tissue ranged from 12 to 30, with very low concentrations in normal tissue. However, the concentration of ^10^B in normal superficial tissue was higher than that in normal brains, and the concentration was between 8 and 15 ppm. This result is consistent with previous studies that have shown that normal brain tissue absorbs boron delivery poorly. But the way they measured it, using the gamma rays of the nuclear reaction of boron, also suggested that the intensity of the gamma rays in the reaction could not be ignored, which is also a factor in tissue damage. Kawabata et al. reported the simultaneous use of sodium boroate and boron-phenylalanine as boron delivery agents, combined with fractional X-ray irradiation. The results showed that the median survival time (MST) for patients treated with BNCT was 15.6 months [95% (CI): 12.2–23.9]; compared with an MST of 10.3 months for patients treated with surgery and external beam radiation therapy (XRT) and chemotherapy (n = 27, MST was 10.3 months, 95% CI: 7.4–13.2), patients treated with BNCT had longer MST.

### Melanoma

Melanoma is a malignant tumor produced by melanocytes, and so far, this remains a difficult cancer to treat with a poor prognosis. The usual treatment for cutaneous melanoma is surgical removal of the focus, sometimes lymph node dissection, and skin graft or flap repair (89–91). **Table 4** summarizes relatively complete clinical studies of BNCT in melanoma from 2004 to date.

**Table 4:** Clinical studies of BNCT application in melanoma.

Institution	Treatment dates	Number of patients	Boron compound and treatment	Clinical outcome	Phase
Comision Nacional de Energia Atomica, Av. Del Libertador (92)	2004	25	BPA-F 14 g/m^2^; 16.5~20 RBE Gy/m^2^		I/II
Instituto de Oncologia Angel H. Roffo. Av. San Martin (40)	2009	7	BPA-F 14 g/m^2^; 13.4~69.3 Gy/m^2^	CR: 69.3%	I/II
The Third Xiangya Hospital of Central South University (18, 93)	2013	22	BPA-F 350 mg/kg; 16.5~20 RBE Gy/m^2^		I/II
National Cancer Center Hospital Recruiting Chuo Ku, Japan (ClinicalTrials.gov Identifier: NCT04293289)	2019	9	BPA 80 mg/kg/h × 2 h; then 40 mg/kg/h	CR: 75%	I/II

BNCT, boron neutron capture therapy; BPA-F, para-boron-phenylalanine-fructose; RBE, relative biological effectiveness; CR, complete response.

González et al. planned to enroll 30 melanoma patients for a clinical trial, and they reported the first results of BNCT for the treatment of melanoma. For the first patient, they administered BPA-F at 14 g/m^2^ for more than 1.5 h, based on the area of the skin at the affected area (94, 95). Results showed that 19 of the 25 nodules were complete response (CR) within 4 weeks of BNCT treatment. After 8 weeks, 21 of the 25 nodules were CR, and 1 was partial response (PR). However, acute Radiation Therapy Oncology Group/European Organisation for Research and Treatment of Cancer (RTOG/EORTC) grade 1 skin reaction was detected on the first day after treatment, but this reaction subsided after 8 weeks (92). Menéndez et al. performed BNCT on seven melanoma patients in Argentina. They injected BPA-F at doses of 14 g/m^2^, with the maximum dose range of 16.5 to 24 Gy/m^2^ for normal skin and 15.8 to 27.5 Gy/m^2^ for normal tissue. The results indicated that the tumor/blood ratio of ^10^B was 1.7 to 2.5 (2 h 45 min for BPA-F injection), 2.4 to 4.1 (5 h for BPA-F injection), and 2.7 to 3.2 (10 h for BPA-F injection). The results suggested that 69.3% of the patients had a response, and the remaining 30.7% had no significant change during the postoperative and follow-up periods. At the same time, ulcerative radiation damage occurred in several patients, which was detected to be caused by high boron concentrations (40).

Yong et al. reported on a BNCT clinical trial in melanoma patients. They injected BPA-F at 350 mg/kg for more than 1.5 h and took samples before and after 1.5 h to calculate boron concentration ratios in different tissues. The results showed that the concentration ratios of T/N, T/B, and N/B were 1.30, 1.93, and 1.49 after 2 h of BPA-F injection, respectively. The concentration ratios of T/N, T/B, and N/B were 1.26, 2.51, and 1.99 after 3 h of BPA-F injection, respectively. After treatment, the lesion was significantly reduced, and no significant radiation damage was observed during follow-up (18). Hiratsuka et al. recently reported long-term clinical results in 8 patients using BPA as a boron delivery agent for BNCT. The experiment was conducted at the Kyoto University Research Reactor (KUR). Patients were irradiated with an epithermal neutron beam within the range of a therapeutic tumor dose and a tolerable skin dose. The results indicated that six of the eight patients had CR, while two patients showed a PR. Of the two patients, another presented with a PR after BNCT treatment and another palindromia after 6 years (96).

### Head and Neck Cancers

Head and neck cancer is one of the most common cancers in the world and is difficult to cure, with high rates in Southeast Asia, Brazil, and central Europe (97, 98). In the past decade, we have learned that it is difficult to eradicate the lesion with a single treatment method, and the selective physical killing of tumor cells by BNCT is expected to be one of the combination methods for clinical treatment of head and neck cancer. BNCT for squamous cell carcinoma of head and neck (SCCHN) and recurrent head and neck carcinoma will be described in this section. **Table 5** summarizes relatively complete clinical studies of BNCT in head and neck cancers from 2007 to date.

**Table 5:** Clinical studies of BNCT application in head and neck cancers.

Institution	Treatment dates	Number of patients	Boron compound and treatment	Clinical outcome	Phase
Helsinki University Central Hospital, Finland (99)	2007	12	BPA-F 400 mg/kg PR: 83%	MST: 12.1 months PR: 83%	I/II
Department of Radiation Oncology, University Hospital Essen (33, 41)	2009	25	BPA 100 mg/kg × 1 h; BSH 50 mg/kg × 1 h	–	I
Department of Oncology, Helsinki University Central Hospital (8, 34, 84)	2012	30	BPA-F 400 mg/kg PR: 76% OS: 30%	MPFS: 7.5 PR: 76% OS: 30%	I/II
Taipei Veterans General Hospital, Taiwan (35)	2011	4	BPA 500 mg/kg; 12~35 Gy/m^2^		I/II
Taipei Veterans General Hospital, Taiwan (36)	2016	17	BPA 400 mg/kg	CR: 67% 2-year OS: 47%	
Taipei Veterans General Hospital, Taiwan (30)	2018	17	BPA-F 180 mg/kg/h	1-year OS: 56%	I/II
Southern Tohoku BNCT Research Center (37)	2021	21	C-BENS with borofalan (^10^B);	CR: 50% (R-SCC) PR: 25% (R-SCC); CR: 8% (R/LA-nSCC) PR: 62% (R/LA-nSCC)	I/II
Taipei Veterans General Hospital, Taiwan (38)	2021	4	BPA-F 180 mg/kg/h	1 patient had a CBS-related death	I/II

BPA-F, para-boron-phenylalanine-fructose; MST, median survival time; PR, partial response; BPA, para-boron-phenylalanine; BSH, borocaptate sodium; CR, complete response; OS, overall survival; C-BENS, cyclotron-based epithermal neutron source; R-SCC, recurrent squamous cell carcinoma; R/LA-nSCC, recurrent and locally advanced non-squamous cell carcinoma.

In 2007, Kankaanranta et al. reported BNCT effects in 12 patients with head and neck cancer. Results showed that 10 of the 12 patients had a PR (PR: 83%), and 2 patients had continued tumor growth. MST was 12.1 months (99). They then re-recruited 30 head and neck cancer patients (29 cancers and 1 sarcoma) for BNCT, 26 of whom received two treatments. The results suggested that of the 29 patients who participated in the efficacy evaluation, 22 patients had a PR (76%), and 6 patients had continued tumor growth. Median progression-free survival (MPFS) was 7.5 months (95% CI: 5.4–9.6 months), and 2-year PFS and OS rates were 20% and 30%, respectively (34). Therefore, it can be seen that BNCT can effectively treat local or recurrent head and neck cancer, and most patients can show a positive response to treatment. Therefore, BNCT can be appropriately considered as a clinical method with chemotherapy and radiotherapy.

Wittig et al. recruited six patients with head and neck cancer and used BSH and BPA as boron delivery agents. The results indicated that both BPA and BSH were better absorbed by the skin and mucous membranes. Therefore, for drug delivery in the patient, the delivery agents need to be overcome in the process of blood circulation by the skin and mucosal consumption. Wittig et al. also discussed the distribution of BSH and BPA in various viscera (32, 33). They suggested that BSH does not preferentially accumulate to liver metastases in patients with colorectal cancer. BPA is preferentially absorbed in liver metastases of colorectal adenocarcinoma to a degree sufficient for BNCT therapy. Therefore, BSH is not suitable as a ^10^B agent for the treatment of liver metastasis. However, the accumulation of BPA in liver metastases to a certain extent can be used to treat the liver *via* BNCT (41).

Wang et al. reported several trials for head and neck cancer. They chose 400 mg/kg of BPA as the administration method. The results indicated that four patients initially recruited showed different degrees of tumor tissue response after treatment, and patients had tolerance (35). In the second study of 17 patients, they also attempted to use BNCT in two sessions, and the results were satisfactory for both primary and recurrent head and neck cancer while maintaining a stable concentration of BPA in the blood (100). Six of the patients presented with a PR to the tumor, five with mucosal inflammation, one with laryngeal edema and carotid hemorrhage, and two with cranial neuropathy. Four patients showed CR (67%), and 2-year OS was 47% (36) in a recent clinical trial involving 17 patients. Wang et al. found that although some patients with BNCT can extend survival to more than 4 years, the inevitable failure factor is a local recurrence, which is difficult to control. At the same time, the complications associated with BNCT can be effectively controlled, which is a gratifying progress. In addition, they proposed that the combination of BNCT with image-guided photon therapy could reduce the recurrence rate and improve the therapeutic efficacy of BNCT (30).

Hirose et al. treated 8 patients with recurrent SCC (R-SCC) and 13 recurrent and locally advanced non-SCC (R/LA-nSCC) patients with cyclotron-based epithermal neutron source (C-BENS) and borofalan (^10^B) *via* BNCT. The objective was to evaluate the efficacy and safety of cyclotron-based BNCT combined with borofalan (^10^B) in the treatment of recurrent or locally advanced head and neck cancer. Results showed that the objective response rate (ORR) for all patients was 71%, and full response/PR was 50%/25% for R-SCC and 8%/62% for R/LA-nSCC. The 2-year OS rates were 58% (R-SCC) and 100% (R/LA-nSCC). The R-SCC median locoregional PFS (LRPFS) was 11.5 months.

Frequently observed adverse events included hair loss (95%), hyperthermia (86%), and nausea (81%), suggesting that BNCT using C-BENS with borofalan (^10^B) is a promising treatment option for head and neck R-SCC or R/LA-nSCC patients (37). Lan et al. performed BNCT on four SCC patients with BPA-F. The results showed that one carotid blowout syndrome (CBS)-related death occurred among patients with signs of CBS, and the others remained recovered until the last follow-up (median follow-up time was 15.1 months) (38).

## Conclusions

Analyzing these clinical results, BNCT may be a good choice for the clinical diagnosis and treatment of cancer. In particular, BNCT has shown remarkable results for problems that are difficult to deal with conventional means. However, the patient situation is generally more complex and prone to various metastases. Secondly, the targeting ability of boron delivery agents also makes it difficult to make the distribution of boron greatly different between tumor and normal tissue. In the occurrence of nuclear reactions, it is inevitable to produce high-energy γ rays that are difficult to ignore, which will easily cause radioactive damage to the surrounding tissue. In addition, due to the relatively small number of patients currently receiving BNCT, long-term observation is still insufficient, and it is difficult to determine the prognostic impact, whether physical or psychological.

## Author Contributions

SW: writing—original draft preparation, investigation, and table and figure preparation. ZZ: investigation and table preparation. LM: investigation. YL: conceptualization, methodology, and supervision. All authors contributed to the article and approved the submitted version.

## Funding

This work was supported by the Special Research Project of Lanzhou University Serving the Economic Social Development of Gansu Province (054000282) Lanzhou Talent Innovation and Entrepreneurship Project (2020-RC-38), the Fundamental Research Funds for the Central Universities (lzujbky-2020-kb14), and Major Science and Technology Special Project of Gansu Province (20ZD7FA003).

## Conflict of Interest

The authors declare that the research was conducted in the absence of any commercial or financial relationships that could be construed as a potential conflict of interest.

## Publisher’s Note

All claims expressed in this article are solely those of the authors and do not necessarily represent those of their affiliated organizations, or those of the publisher, the editors and the reviewers. Any product that may be evaluated in this article, or claim that may be made by its manufacturer, is not guaranteed or endorsed by the publisher.
